# Female genital mutilation and intimate partner violence in the Ivory Coast

**DOI:** 10.1186/1472-6874-14-13

**Published:** 2014-01-22

**Authors:** Karl Peltzer, Supa Pengpid

**Affiliations:** 1 ASEAN Institute for Health Development, Mahidol University, Salaya, Phutthamonthon 73170, Thailand; 2 Department of Psychology, University of Limpopo, Turfloop Campus, Private Bag X1106, Sovenga 0727, South Africa; 3 HIV/AIDS, STIs and TB (HAST), Human Sciences Research Council, Private Bag X41, Pretoria 0001, South Africa

**Keywords:** Female genital mutilation, Intimate partner violence, Risk factors, Women, Ivory Coast

## Abstract

**Background:**

Serious forms of violence against women include Female Genital Mutilation (FGM) and Intimate Partner Violence (IPV). The aim of this study was to determine if FGM is associated with IPV, using data obtained from the Demographic and Health Survey (DHS) 2012 in Ivory Coast.

**Methods:**

Participants for this study were drawn from the 2011-12 Ivory Coast Demographic and Health Survey (CDHS), a nationally representative sample of 10060 women aged 15 to 49 years. The analysis of this paper is restricted to the sample of women who responded to the FGM and domestic violence modules (N = 5005).

**Results:**

The lifetime prevalence of physical violence was 24.8%, sexual violence, 5.7%, and emotional violence, 19.0%, and the prevalence of any lifetime IPV was 32.1%. In all, 40.6% reported female genital cutting or mutilation (FGM). Women reporting FGM were two times as likely to experience sexual IPV (AOR: 1.96, CI: 1.29-2.98), while other subtypes of IPV were higher in women reporting FGM but they were not significant. Of the socio-demographic covariates, urban residence and having a primary education were associated with most subtypes of IPV, while being a Muslim seemed protective from any type, sexual and emotional IPV. Having seen the father beating the mother was positively associated with most IPV subtypes, and having been diagnosed with a sexually transmitted infection (STI) in the previous 12 months was associated with physical and sexual IPV.

**Conclusion:**

Significant rates of FGM and IPV were found among this sample of Ivorian women calling for the need for multiple strategies to reduce FGM and IPV.

## Background

Female genital mutilation (FGM) – defined by the World Health Organization (WHO) and the United Nations (UN) agencies as “the partial or total removal of the female external genitalia or other injury to the female genital organs for non-medical reasons” is a deeply rooted tradition in many communities in 28 countries in Africa and in some countries in Asia and the Middle East [1], ranging from 0.6% to 98% of the female population [WHO 2011]. Across countries in western Africa the prevalence of Female Genital Cutting (FGC) was 94%, 79%, 74% and 72% in Sierra Leone, Gambia, Burkina Faso and Mauritania, respectively, whereas in Ghana, Niger and Togo prevalence was less than 6% [2]. In Ivory Coast a comparison between Multiple Indicator Cluster Survey (MICS) 2000 and MICS 2006 revealed that between 2000 and 2006 the national prevalence of FGM among women aged 15-49 decreased from 44% to 36.4% [3]. Various short-term and long term negative physical and psychosocial health consequences of FGM have been reported [1,4]. Some examples of these health consequences include adverse effects on women’s reproductive health (prolonged labour, obstetric lacerations, instrumental delivery, obstetric hemorrhage, and difficult delivery) [4] and negative psychological consequences (have a psychiatric diagnosis, suffer from anxiety, somatisation, phobia, and low self-esteem) [5].

According to WHO [6] the global prevalence of physical and/or sexual intimate partner violence among all ever-partnered women was 30.0%, with the highest in the WHO African, Eastern Mediterranean and South-East Asia Regions, where approximately 37% of ever-partnered women reported having experienced physical and/or sexual intimate partner violence at some point in their lives [6]. From the 2005 Demographic and Health Survey in Ivory Coast it was found that past 12 months physical and/or sexual violence from an intimate partner or non-partner was 12% [7].

Two previous studies in Egypt and Mali have shown a positive association between FGM and intimate partner violence (IPV) [8,9]. Salihu et al. [8] propose that a possible explanation of the association between FGM and IPV is that women with previous exposure to violence are more likely to experience violence later in life. Women with physical and sexual trauma during childhood are more likely to experience violence, including IPV in later life [10]. Likewise, women who experience FGM as a form of violence in early childhood may be vulnerable to IPV [8]. Childhood abuse, including FGM may interfere with normal development of interpersonal relatedness and affect regulation leading to a “higher threshold of tolerance” for coercive or forceful sexual advances [11]. The aim of this study was to determine if FGM is associated with IPV, using data obtained from the Demographic and Health Survey (DHS) 2012 in Ivory Coast.

## Methods

### Sample and procedure

Participants for this study were drawn from the 2011-12 Ivory Coast Demographic and Health Survey (CDHS), a nationally representative sample of 10060 women aged 15 to 49 years. The 2011/12 CDHS employed a two-stage stratified sample, where systematic sampling with probability proportional to size was applied [12]. The analysis of this paper is restricted to the sample of women who responded to the FGM and domestic violence modules (N = 5005). This study is based on analysis of secondary data with all participant identifiers removed. Permission to use the DHS data in this study was obtained from Opinion Research Corporation (ORC) Macro Inc. The DHS data are publicly available. The survey procedure and instruments used have received ethical approval from the National Ethics Committee of Côte d’Ivoire and the Internal Review Board (IRB) of the Centers of Disease Control (CDC) in Atlanta [12].

### Measures

The questionnaire included demographic variables such as age, formal education, work status, residence, marriage type, religious denomination and wealth status, a composite index based on the household’s ownership of consumer items such as television, car, drinking water, toilet facilities, etc.

*Female Genital Mutilation* (FGM) was assessed from the survey item ‘Respondent Circumcised,’ which was a dichotomous (Yes/No) variable. Further, those who indicated that they had been circumcised were asked several items regarding circumcision, including ‘flesh removed from the genital area’, ‘genital area just nicked without removing any flesh’ and ‘Genital area sewn closed’ and the timing of circumcision [12].

#### *Intimate partner violence* (IPV)

The domestic violence module of the Ivory Coast DHS includes 11 items that capture violence committed by a male partner or spouse. From these questions, IPV was categorised into three main subtypes: physical, sexual and emotional. Physical violence referred to any exposure to one or several of the following acts against women by a current or former husband or partner ever: i) pushing, shaking or throwing something at her; ii) slapping her or twisting her arm; iii) punching or hitting her with something harmful; iv) kicking or dragging her; v) strangling or burning her; vi) threatening her with a weapon (e.g. Gun or knife); and vii) attacking her with a weapon. Sexual violence referred to any exposure to one or several of the following acts against women by a current or former husband or partner ever: i) forced sexual intercourse; and ii) other sexual acts when undesired. Emotional IPV was assessed with two items, ‘ever humiliation’ and ‘ever threatened harm’, and was defined as a woman’s report of ever experiencing an act of emotional violence by a partner. Exposure to each of these types of violent acts were scored as 1 (any experience of violence ever) and 0 (no experience of violence ever).Women were further categorised as those who experienced one type of IPV and those who experienced two or more types of IPV [8,9].

In addition, several risk factors for IPV were assessed, including increased risk of HIV/STI [6] and witnessing IPV of parents in childhood [13,14].

### Data analysis

Data analysis was performed using STATA software version 11.0 (Stata Corporation, College Station, Texas, USA). The analysis in STATA took into account the multilevel stratified cluster sample design of the study. Frequencies as estimation of prevalence of IPV were obtained. Logistic regression analysis was conducted to estimate the association between relevant predictor variables including FGM and IPV. Adjusted odds ratios are reported for selected predictor variables (FGM, age, formal education, residence, religious affiliation, wealth status, working status, marriage type, history of an STI, parental violence) while considering IPV as a dependent variable. In the analysis, weighted percentages are reported. The reported sample size refers to the sample that was asked the target question. The two-sided 95% confidence intervals are reported. The p-value less or equal to 5% is used to indicate statistical significance. Both the reported 95% confidence intervals and the p-value are adjusted for the multi-stage stratified cluster sample design of the study.

## Results

Overall, 5005 women were administered the domestic violence and female genital cutting modules of the 2012 Ivory Coast DHS. Their demographic characteristics are provided in Table 1. The lifetime prevalence of physical violence was 24.8%, sexual violence, 5.7% and emotional violence, 19.0%, and the prevalence of any lifetime IPV was 32.1%. In all, 40.6% reported female genital cutting or mutilation (FGM). Among women with FGM the median age of circumcision was 4 years (SD = 5.4). The proportion of women with FGM was highest among those with no education (55.8%), those belonging to the Catholic religion (71.8%), and women in polygynous marriage (59.5%) (see Table 1).

**Table 1:** Sample characteristics of Ivory Coast women completing the domestic violence and female genital cutting modules of the Demographic and Health Survey, 2012

	**Total**	**FGM**	**Any type of IPV**	**Physical IPV**	**Sexual IPV**	**Emotional IPV**	**One type of IPV**	**Two or three types of IPV**
	**N (%)**	**%**	**%**	**%**	**%**	**%**	**%**	**%**
All	5005	40.9	31.2	24.8	5.7	19.0	16.5	14.7
Age (years)								
15-24	1177 (21.9)	36.3	30.6	25.5	6.5	17.0	16.1	14.5
25-34	2096 (40.9)	40.4	30.6	24.3	5.1	19.0	16.5	14.1
35-49	1732 (37.3)	48.0	32.3	25.0	5.9	20.4	16.8	13.5
Education								
None	3392 (63.0)	54.8	28.9	23.0	4.4	16.8	16.7	12.2
Primary	1105 (24.3)	29.4	37.5	29.8	9.5	23.6	17.2	20.4
Secondary or higher	508 (12.7)	20.6	30.6	24.5	5.0	21.7	14.2	16.4
Religion								
Catholic	2317 (42.6)	67.4	30.4	25.3	4.5	17.1	16.8	13.6
Muslim	849 (17.0)	19.1	32.2	24.3	6.0	21.3	16.0	16.2
Protestant/other Christian	1028 (25.3)	14.0	33.0	25.6	7.6	21.2	16.3	16.7
Animist/no/other	742 (15.2)	45.9	30.0	22.9	6.0	19.3	16.7	13.4
Marriage type								
Monogamous	3436 (71.2)	43.6	29.6	23.8	5.3	17.8	15.6	14.0
Polygynous	1155 (28.8)	59.5	32.7	25.6	6.3	19.4	18.5	14.2
Wealth								
Poorest	1162 (21.4)	41.7	27.8	20.5	6.4	15.6	16.6	11.1
Second	1076 (19.9)	36.9	29.7	24.7	5.1	18.3	15.0	14.7
Middle	1063 (19.3)	50.0	31.1	24.0	4.7	19.0	17.4	13.6
Fourth	965 (20.5)	45.3	34.9	28.1	7.2	21.5	18.6	16.3
Richest	739 (19.0)	32.5	32.8	27.1	4.9	21.2	14.7	18.1
Residence								
Rural	3167 (57.8)	42.3	29.0	22.4	5.3	16.7	16.9	12.1
Urban	1838 (42.2)	39.5	34.3	28.2	6.3	22.2	16.0	18.3
Working status								
Not working	1277 (26.0)	35.3	29.2	23.6	5.4	16.7	16.5	12.7
Currently working	3715 (74.0)	43.6	32.0	25.3	5.8	19.9	16.6	15.4
Father beat mother	643 (14.7)	47.1	43.9	35.3	8.4	24.1	24.9	18.9
Had STI in the past 12 months	324 (6.6)	45.7	38.2	33.4	9.2	22.5	19.3	18.9

The adjusted odds ratios (AOR) and 95% confidence intervals (95% CI) for the association between FGM and IPV are shown in Table 2. Women reporting FGM were two times as likely to experience sexual IPV (AOR: 1.96, CI: 1.29-2.98), while other subtypes of IPV were higher in women reporting FGM but they were not significant. Of the socio-demographic covariates, urban residence and having a primary education were associated with most subtypes of IPV, while being a Muslim seemed protective from any type, sexual and emotional IPV, and having been exposed to two or three types of IPV. Having seen the father beating the mother was positively associated with most IPV subtypes, and having been diagnosed with a sexually transmitted infection (STI) in the previous 12 months was associated with physical and sexual IPV (see Table 2).

**Table 2:** Adjusted odds ratios and 95% confidence intervals for the association between female genital mutilation, other variables and intimate partner violence and its subtypes

	**Any type of IPV AOR (95% CI)**	**Physical IPV AOR (95% CI)**	**Sexual IPV AOR (95% CI)**	**Emotional IPV AOR (95% CI)**	**One type of IPV AOR (95% CI)**	**Two or three types of IPV AOR (95% CI)**
FGM	1.00	1.00	1.00	1.00	1.00	1.00
	1.26 (0.98-1.63)	1.15 (0.87-1.51	1.96 (1.29-2.98)**	1.18 (0.88-1.59)	1.30 (0.99-1.69)	1.13 (0.80-1.58)
Age (years)						
15-24	1.00	1.00	1.00	1.00	1.00	1.00
25-34	1.02 (0.80-1.31)	0.96 (0.75-1.24)	0.75 (0.47-1.19)	1.08 (0.77-1.51)	1.12 (0.84-1.49)	0.91 (0.65-1.29)
35-49	1.06 (0.82-1.36)	0.95 (0.74-1.22)	0.92 (0.59-1.44)	1.14 (0.79-1.65)	1.04 (0.76-1.43)	1.05 (0.73-1.51)
Education						
None	1.00	1.00	1.00	1.00	1.00	1.00
Primary	1.42 (1.11-1.82)**	1.40 (1.07-1.84)*	1.91 (1.18-3.10)**	1.43 (1.06-1.92)*	1.01 (0.75-1.35)	1.75 (1.26-2.43)*
Secondary or higher	0.93 (0.64-1.35)	0.91 (0.59-1.41)	1.25 (0.57-2.75)	1.01 (0.64-1.61)	0.92 (0.58-1.47)	0.98 (0.57-1.66)
Religion						
Catholic	1.00	1.00	1.00	1.00	1.00	1.00
Muslim	0.75 (0.57-0.99)*	0.86 (0.64-1.16)	0.51 (0.28-0.95)*	0.63 (0.45-0.89)**	0.95 (0.68-1.33)	0.65 (0.45-0.94)*
Protestant/other Christian	0.99 (0.76-1.31)	0.96 (0.72-1.28)	1.22 (0.76-1.97)	1.00 (0.68-1.46)	1.03 (0.74-1.43)	0.97 (0.67-1.41)
Animist/no/other	0.96 (0.69-1.32)	0.97 (0.68-1.40)	0.84 (0.42-1.68)	1.12 (0.74-1.70)	0.97 (0.65-1.43)	0.99 (0.65-1.52)
Marriage type						
Monogamous	1.00	1.00	1.00	1.00	1.00	1.00
Polygynous	1.19 (0.92-1.53)	1.11 (0.87-1.42)	1.39 (0.80-2.43)	1.16 (0.88-1.52)	1.21 (0.88-1.66)	1.08 (0.79-1.48)
Wealth						
Poorest	1.00	1.00	1.00	1.00	1.00	1.00
Second	0.87 (0.66-1.15)	1.04 (0.74-1.45)	0.66 (0.33-1.34)	0.95 (0.68-1.33)	0.76 (0.53-1.08)	1.07 (0.74-1.56)
Middle	1.01 (0.68-1.51)	1.04 (0.69-1.57)	0.57 (0.24-1.35)	1.03 (0.73-1.45)	1.03 (0.63-1.69)	0.95 (0.62-1.46)
Fourth	1.05 (0.69-1.61)	1.05 (0.66-1.68)	0.64 (0.21-1.94)	0.99 (0.64-1.54)	1.20 (0.73-1.96)	0.85 (0.48-1.48)
Richest	0.92 (0.55-1.55)	1.07 (0.61-1.86)	0.60 (0.19-1.89)	0.82 (0.46-1.45)	0.87 (0.47-1.64)	0.95 (0.48-1.87)
Residence						
Rural	1.00	1.00	1.00	1.00	1.00	1.00
Urban	1.43 (1.00-2.05)*	1.43 (1.00-2.05)*	1.69 (0.73-3.93)	1.77 (1.13-2.77)*	1.00 (0.69-1.45)	1.94 (1.22-3.09)**
Working status						
Not working	1.00	1.00	1.00	1.00	1.00	1.00
Currently working	1.12 (0.88-1.43)	1.13 (0.88-1.44)	1.00 (0.64-1.56)	1.18 (0.89-1.56)	1.00 (0.74-1.35)	1.23 (0.88-1.71)
Father beat mother	1.80 (1.37-2.37)***	1.88 (1.43-2.49)***	1.58 (0.94-2.67)	1.40 (0.98-1.99)	1.57 (1.13-2.16)*	1.61 (1.15-2.27)**
Had STI in the past 12 months	1.45 (1.02-2.05)*	1.67 (1.14-2.45)**	1.84 (1.02-3.29)*	1.22 (0.79-1.89)	1.25 (0.75-2.09)	1.45 (0.91-2.30)

***P < 0.001; **P < 0.01; *P < 0.05.

## Discussion

The lifetime prevalence of IPV and its subtypes in the Ivory Coast seem lower than the average in the WHO African region [6]. The prevalence of FGM found in this survey (40.6%) seems to show an increase compared to 36.4% in 2006 [3].

The study findings show that FGM was only partially associated with IPV, namely with sexual violence only, while having witnessed interparental violence in childhood was found to be associated with most IPV subtypes; the latter was also found in a DHS survey in Bolivia [14]. Therefore, our results are only partially consistent with previous research that links childhood exposure to physical or sexual trauma (e.g. FGM) to subsequent IPV [8,13,15,16]. Previous studies found that FGM women had higher marital and sexual relationship dissatisfaction than non-FGM women [17]. Further research is needed to explore the reasons for the identified association between FGM and sexual violence in this study.

Further, the study found that having been diagnosed with a sexually transmitted infection (STI) in the previous 12 months was associated with physical and sexual IPV. IPV has been identified to increase the risk of HIV/STI [6]. Of the socio-demographic covariates studied, urban residence and having a primary education were associated with most subtypes of IPV, while being a Muslim seemed protective from any type, sexual and emotional IPV and multiple types of IPV. These findings seem to be in agreement with other studies in terms of urban residence [14] and better education [13]. Further, our analysis of the correlates of IPV showed that physical, psychological and sexual violence had a number of common risk factors, as also found by Meekers et al. [14] among Bolivian women.

### Study limitations

Caution should be taken when interpreting the results of this study due to certain limitations. Since this was a cross-sectional study, causality between the compared variables cannot be concluded. A further limitation was that some factors known to be contributing to IPV were not assessed [13].

## Conclusions

Significant rates of FGM and IPV were found among this sample of Ivorian women calling for the need for multiple strategies to reduce FGM and IPV.

## Competing interests

The authors declare that they have no competing interests.

## Authors’ contributions

KP performed the analysis and drafted the manuscript. SP developed the idea of the analysis and provided comments on the manuscript. All authors read and approved the final manuscript.

## Pre-publication history

The pre-publication history for this paper can be accessed here:

http://www.biomedcentral.com/1472-6874/14/13/prepub
